# Molecular epidemiology of *Neisseria gonorrhoeae* isolates in Russia, 2015–2023: current trends and forecasting

**DOI:** 10.3389/fcimb.2025.1526859

**Published:** 2025-02-19

**Authors:** Ilya Kandinov, Boris Shaskolskiy, Dmitry Kravtsov, Anatoliy Larkin, Alexei Kubanov, Marina Shpilevaya, Julia Shagabieva, Nikita Nosov, Dmitry Gryadunov

**Affiliations:** ^1^ Center for Precision Genome Editing and Genetic Technologies for Biomedicine, Engelhardt Institute of Molecular Biology, Russian Academy of Sciences, Moscow, Russia; ^2^ State Research Center of Dermatovenerology and Cosmetology, Russian Ministry of Health, Moscow, Russia

**Keywords:** *Neisseria gonorrhoeae*, molecular epidemiology, antibiotic resistance, genetic determinants of drug resistance, genotyping

## Abstract

**Introduction:**

The emergence of multidrug resistance in *N. gonorrhoeae* is a serious global problem, and gonorrhea may soon become an incurable disease. The aim of the study was to characterize the *N. gonorrhoeae* population in Russia from 2015 to 2023 and predict the potential spread of the most concerning clones.

**Methods:**

A total of 996 N*. gonorrhoeae* isolates were examined during the analyzed period. Ceftriaxone and azithromycin susceptibility testing were performed using the agar dilution method. Microarray-based assays and sequencing were employed to identify the genotypes and genetic markers of antimicrobial resistance.

**Results:**

No ceftriaxone-resistant isolates were found in Russia, however, the number of isolates with reduced susceptibility to ceftriaxone has increased to 22.6% in recent years. Since 2020, approximately 12.5% of isolates have exhibited resistance to azithromycin annually. Two clusters of isolates pose a particular threat to Russia: NG-MAST G2212, linked to MLST 1901/1902, carries a mosaic structure in the *penA* gene; G12302, linked to MLST 9363, contains mosaic alleles in the *mtrR* and *mtrD* genes. Additionally, two new high-risk genogroups were characterized: G18898 and G16206. Both are associated with MLST 10314 and harbor mosaic variants of *penA* or *mtrR*/*mtrD*. Analysis of time series data suggests that isolates with mosaic alleles are unlikely to be eradicated from the population in the near future, potentially worsening the epidemiological situation of gonorrhea in Russia.

**Conclusions:**

The native genetic strains of *N. gonorrhoeae* in Russia, which are susceptible to cephalosporins and macrolides, are being progressively replaced by globally dominant lineages. To further characterize this epidemiologic shift, ongoing surveillance strategies using molecular epidemiology and the identification of genetic markers will be crucial in curbing the growth and spread of *N. gonorrhoeae* resistance. Such efforts are vital in ensuring the availability of effective treatments for gonococcal infection.

## Introduction

Gonorrhea is one of the most prevalent sexually transmitted infections. According to the World Health Organization, approximately 82 million cases of gonorrhea are reported globally each year (WHO, 2024a). In recent years, the incidence of gonorrhea has risen in countries such as China, the United States, and several European nations. An exceptional feature of the *Neisseria gonorrhoeae* pathogen is its rapid development of resistance to the antimicrobial drugs used in its treatment, which has repeatedly necessitated modifications to current treatment regimens and higher therapeutic doses (WHO, 2024b; Barbee and St Cyr, 2022). Particularly concerning is the emergence and widespread dissemination of multidrug-resistant *N. gonorrhoeae* clones, including strains resistant to current first-line treatments such as third-generation cephalosporins and macrolides. This increases the risk of the potential emergence of untreatable gonorrhea in the near future (Fifer et al., 2024; Ouk et al., 2024; Unemo et al., 2024).

In recent years, there has been a slight upward trend in the incidence of gonorrhea in the Russian Federation, with rates of 8.1 and 7.8 cases per 100,000 population in 2022 and 2023, respectively (Kubanov and Bogdanova, 2024; Shagabieva et al., 2023). Although there have been no reports of cephalosporin-resistant isolates to date, recent cross-border transmission has led to an increase in the proportion of macrolide-resistant isolates. This raises concerns about the future viability of combined antimicrobial therapy (ceftriaxone + azithromycin) in Russia, similar to the regimen recommended in the United States and several Western European countries (Kandinov et al., 2023a, 2023). Given the global spread of drug-resistant gonococcal clonal lineages, these trends underscore the importance of monitoring *N. gonorrhoeae* transmission and antimicrobial resistance within Russia.

Modern recommendations for the control of gonococcal infections include molecular genotyping protocols aimed at preventing transmission within populations and timely detection of drug-resistant genotypes (European Centre for Disease Prevention and Control, 2024). Currently in Russia, as in many other countries, *N. gonorrhoeae* isolates are monitored using genotyping systems such as MLST (Multilocus Sequence Typing) and NG-MAST (Neisseria gonorrhoeae Multi-Antigen Sequence Typing) (Shaskolskiy et al., 2020; Kandinov et al., 2023a). The whole-genome sequencing (WGS) and the cgMLST protocol offer the most comprehensive insights, including molecular genotyping and identification of genetic resistance determinants (Harrison et al., 2020). However, the relatively high cost of WGS and the personnel skill requirements for genome assembly and result processing remain barriers to the pervasive use of genome-wide sequencing for clinical isolates.

At the Engelhardt Institute of Molecular Biology, Russian Academy of Sciences, hydrogel-based biological microarrays have been developed and successfully applied in clinical practice (Gryadunov et al., 2018; Shaskolskiy et al., 2021a). These microarrays provide rapid and efficient detection of selected nucleotide polymorphisms in DNA samples. Microarray-based assays have been designed to identify genetic determinants of resistance to key antimicrobial drugs for gonorrhea treatment, namely ceftriaxone and azithromycin (Shaskolskiy et al., 2021b; Kandinov et al., 2023a). Additionally, a microarray has been developed to detect 18 informative polymorphisms in genes used for MLST typing, providing relatively rapid and cost-effective genotyping of a large number of samples of *N. gonorrhoeae* clinical isolates (Kandinov et al., 2024).

Using these assays, clones of Russian isolates carrying the *penA* allele type 34.001, along with mutations in the *ponA* and *porB* genes, have been identified. These isolates exhibited reduced susceptibility to third-generation cephalosporins and belonged to the pandemically dangerous genogroup NG-MAST 1407 (Kandinov et al., 2020; Shaskolskiy et al., 2021b). More recently, due to the cross-border transmission of the European NG-MAST 12302 genogroup into Russia, there has been a sharp increase in the proportion of azithromycin-resistant isolates carrying the mosaic promoter (meningitidis-like promoter) of the *mtrR* gene and the mosaic sequence of the *mtrD* gene (Kandinov et al., 2023a).

However, the general trends in the evolution of the modern *N. gonorrhoeae* population in Russia remain unclear. Data on the current MLST genotypes of *N. gonorrhoeae* circulating in Russia are not fully available in the literature, nor is there sufficient information on the evolution of molecular types and genetic determinants of resistance. The lack of a comprehensive understanding of the Russian *N. gonorrhoeae* population may result in outdated clinical recommendations, potentially leading to an increase in gonococcal infections due to the ineffectiveness of current antimicrobial treatments.

The aim of this study was to characterize the *N. gonorrhoeae* population in Russia from 2015 to 2023, including data on antimicrobial susceptibility and genetic determinants of resistance to ceftriaxone and azithromycin. Additionally, the study aimed to assess biodiversity based on NG-MAST and MLST genotyping. The objectives also included forecasting the distribution of isolates with mosaic *penA*, *mtrR*, and *mtrD* genes to better understand the processes shaping the current *N. gonorrhoeae* population in Russia.

## Materials and methods

### Collection and characterization of Russian *N. gonorrhoeae* isolates

Clinical isolates of *N. gonorrhoeae* collected in Russia were obtained from the State Scientific Centre of Dermatovenerology and Cosmetology of the Ministry of Health of the Russian Federation. Isolates were collected, analyzed, and stored as previously described (Kubanov et al., 2019; Shaskolskiy et al., 2019). Samples from the urethra in males and cervix/urethra in females (each sample from one patient) were seeded onto chocolate blood agar supplemented with 1% IsoVitaleX enrichment and 1% VCAT selective additive (vancomycin, colistin, amphotericin, and trimethoprim) (bioMérieux, Marcy l’Etoile, France). Primary identification of *N. gonorrhoeae* included Gram staining and rapid oxidase reaction. Complete identification of *N. gonorrhoeae* was based on the sugar utilization test using the NH identification card for the VITEK 2 Compact analyser (bioMérieux, Marcy l’Etoile, France) and MALDI-TOF MS using a MALDI Biotyper (Bruker Daltonics, Bremen, Germany).

During the period from 2015 to 2023, samples were received each year from at least 18 participating regions geographically distributed across Russia. In total, 996 samples were collected and analyzed during the study period: 2015 – 123, 2016 – 253, 2017 – 127, 2018 – 150, 2019 – 119, 2020 – 116, 2021 – 46, and 2022–2023 – 62. The isolates collected in 2022–2023 were pooled to maintain the sampling balance, which had been disrupted by the COVID-19 pandemic. The *N. gonorrhoeae* isolates were stored at -70°C and used for DNA isolation and phenotypic susceptibility analyses.

### *N. gonorrhoeae* antimicrobial susceptibility testing

Ceftriaxone and azithromycin susceptibility testing of *N. gonorrhoeae* isolates and determination of the MIC were carried out using the agar dilution method. The obtained MIC values were compared with breakpoints, including ECOFF parameters from The European Committee on Antimicrobial Susceptibility Testing (EUCAST), 2024. For ceftriaxone, isolates with an MIC_cro_ of ≤ 0.125 mg/L were considered susceptible, those with MIC_cro_ ≤ 0.06 mg/L were considered decreased susceptible, and isolates with MIC_cro_ > 0.125 mg/L were considered resistant. For azithromycin, isolates with MIC_azi_ ≤ 1 mg/L were considered susceptible, and those with MIC_azi_ > 1 mg/L were considered resistant.

### Identification of genetic determinants of *N. gonorrhoeae* resistance to ceftriaxone and azithromycin and genotyping using microarray technology

Clinical isolates of *N. gonorrhoeae* were analyzed using previously designed hydrogel-based microarrays. The microarrays were produced by copolymerization immobilization technique according to a procedure developed earlier (Shaskolskiy et al., 2021a). Mutations in the *penA*, *ponA*, and *porB* genes were detected using a microarray developed for analyzing genetic determinants of resistance to third-generation cephalosporins (ceftriaxone) (Shaskolskiy et al., 2021a). The analysis targeted the following gene codons: *penA* (311, 312, 316, 345-346, 501, 512, 542, 545, 551), *ponA* (421), and *porB* (120 and 121).

Mutations in the promoter region of the *mtrR* gene (positions -35 and -10), mutations in the *mtrD* gene (codons 821 and 823), and mutations in the 23S rRNA gene of all four copies (position 2611) were detected using a previously developed microarray designed to analyze genetic determinants of resistance to macrolides (azithromycin) (Kandinov et al., 2023a). For the initial mass identification of MLST genotypes, we used another microarray along with the miniMLST typing scheme (Kandinov et al., 2024); results were subsequently verified using classical genotyping methods, including Sanger sequencing.

### Genotyping of Russian *N. gonorrhoeae* isolates using NG-MAST and MLST schemes

Molecular typing of *N. gonorrhoeae* isolates was performed using standard NG-MAST and MLST protocols. The internal regions of the *porB* and *tbpB* genes (NG-MAST) and the *abcZ*, *adk*, *aroE*, *fumC*, *gdh*, *pdhC*, and *pgm* genes (MLST) were subjected to PCR amplification. The resulting products were purified and sequenced. Allele numbers for the two NG-MAST sequences and seven MLST sequences were assigned according to the pubMLST database (https://pubmlst.org). NG-MAST and MLST sequence type numbers were obtained for each isolate. A complete list of all isolates and their corresponding sequence type numbers is presented in **Supplementary Table S1**.

Phylogenetically related genogroups were identified for all the NG-MAST types. Genogroups were defined as described previously (Shaskolskiy et al., 2020) as a set of *porB* and *tbpB* alleles (variable internal regions), where the concatenated sequence of both alleles (880 bp) differed by no more than 5 nucleotides from the concatenated sequence of both alleles of the major sequence type (ST) in the genogroup. Allele similarity was assessed using the MEGA X software. Major genogroups were defined as those containing at least 1% of the total sample of isolates (996 isolates).

### Constructing a phylogenetic tree (phylogram)

A multiple sequence alignment of nine concatenated loci from the *abcZ*, *adk*, *aroE*, *fumC*, *gdh*, *pdhC*, *pgm*, *porB*, and *tbpB* genes used for MLST and NG-MAST genotyping was generated, resulting in 376 unique sequences after removal of duplicates. A maximum likelihood phylogenetic tree was constructed using RAxML software (version 8.2.4; https://usegalaxy.eu), with 1000 rapid bootstrap inferences. The phylogenetic tree was visualized using FigTree v1.4.4 software (http://tree.bio.ed.ac.uk).

### A statistical analysis and forecasting of the frequencies of isolates with mosaic *penA*, *mtrR*, and *mtrD* genes

The frequencies of mosaic genes were treated as time series. To analyze the time series and make predictions using ARIMA models, the guide by Hyndman and Athanasopoulos (2018) was used. Calculations were performed in R using the following libraries: fpp2 (version 2.5), tseries (version 0.10-58), and forecast (version 8.23).

Stationarity of the series was assessed using the KPSS test and the Augmented Dickey-Fuller test. If necessary, the lambda parameter for the Box-Cox transformation was calculated. Differentiation was carried out based on the required number of iterations, and model parameters were selected using the AICc criterion. For the resulting model, residuals were examined by plotting the autocorrelation function (ACF) of the residuals and performing a portmanteau test of the residuals.

## Results

### Ceftriaxone and azithromycin susceptibility testing

**Figure 1:**
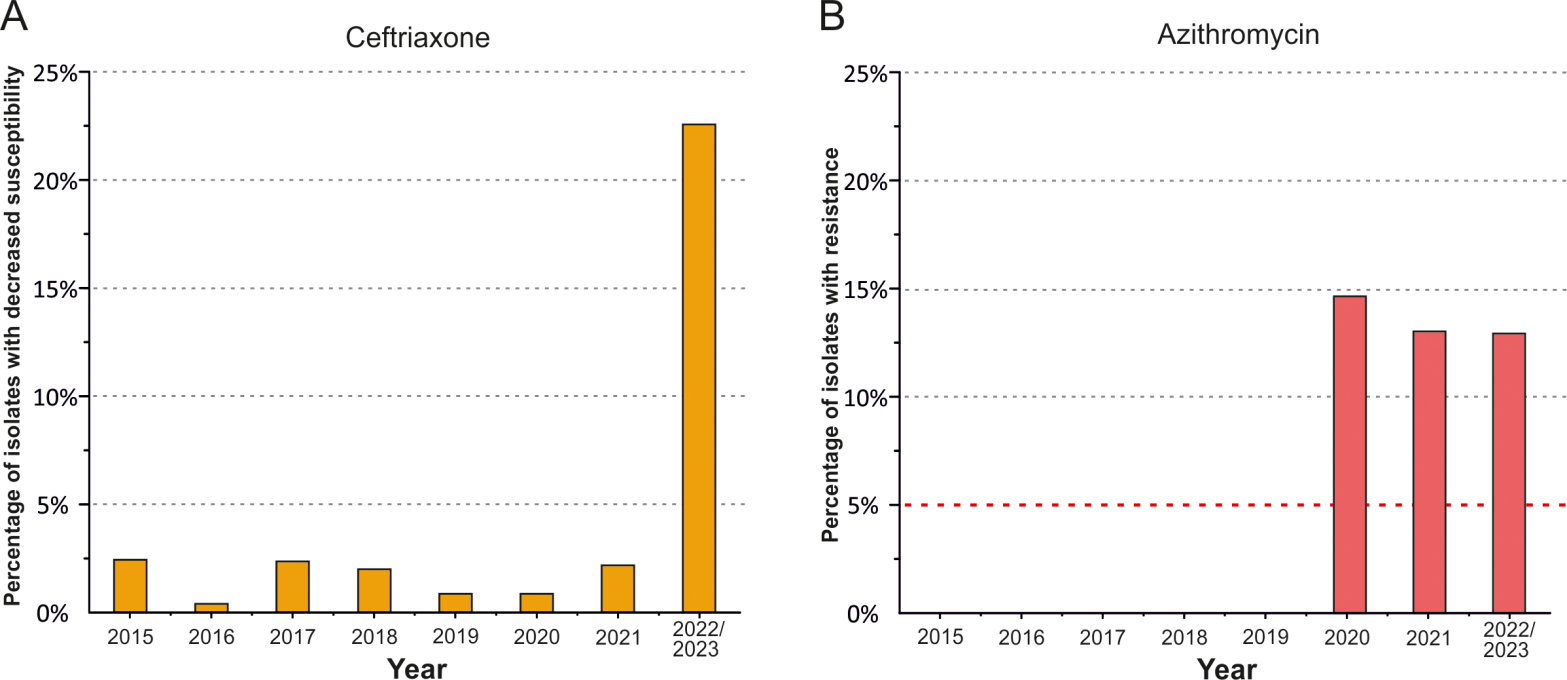
Diagram of the distribution of *N. gonorrhoeae* isolates in the Russian population (2015-2023). **(A)** Proportion of isolates with reduced susceptibility to ceftriaxone (MIC_cro_ ≥ 0.06 mg/L). **(B)** Proportion of isolates resistant to azithromycin (MIC_azi_ > 1 mg/L). The red dotted line in panel **(B)** represents the 5% threshold, above which the WHO guidelines no longer recommend azithromycin for the treatment of gonococcal infection.

A total of 996 *N. gonorrhoeae* isolates were sequentially collected and tested for MICs of ceftriaxone and azithromycin (**Supplementary Table S1**). The MIC range for ceftriaxone was 0.002–0.12 mg/L, while the MICs for azithromycin ranged from 0.02 mg/L to 8 mg/L. The proportions of isolates with reduced susceptibility to ceftriaxone and resistance to azithromycin detected in Russia during the period from 2015 to 2023 are shown in **Figure 1**.

During the analyzed time period, no ceftriaxone-resistant isolates were detected, confirming the continued suitability of third-generation cephalosporins for the treatment of gonococcal infections in Russia. As shown in **Figure 1A**, the proportion of isolates with decreased susceptibility to ceftriaxone did not exceed 1–2% per year from 2015 to 2021. However, in 2022–2023, 22.6% of isolates exhibited decreased susceptibility. This indicates a notable increase in the proportion of isolates with reduced susceptibility to ceftriaxone in the Russian population in recent years, which may create conditions for the emergence of resistant isolates in the Russian Federation in the near future.

All isolates collected from 2015 to 2019 were susceptible to azithromycin (**Figure 1B**). However, in 2020, a sharp increase in azithromycin-resistant isolates was observed, reaching 14.7% in the Russian population. In the subsequent period, from 2021 to 2023, the proportion of azithromycin-resistant isolates remained relatively high, at around 13%, more than double the WHO-recommended threshold of 5% for the use of antibiotic.

### Genetic determinants of resistance to ceftriaxone and azithromycin in the Russian population of *N. gonorrhoeae*

**Table 1** presents data on the distribution of genetic determinants of resistance to ceftriaxone and azithromycin in *N. gonorrhoeae* isolates from the Russian population between 2015 and 2023. As seen in **Table 1**, the proportion of determinants in the Russian population of *N. gonorrhoeae* changes from year to year. Since 2015, there has been an increase in the number of isolates with the substitutions Ile312Met, Val316Thr, Ala501Val, Asn512Tyr, Gly542Ser, and Gly545Ser, some of which are associated with an increase in the mosaicism of *penA* to 11% in 2022-2023 (**Supplementary Table S1**). Additionally, the determinants Ala501Thr and Pro551Leu are gradually being eliminated and replaced in the population. The observed changes in *penA*, along with *ponA*, *porB*, *mtrR*, and other unknown mechanisms, may have led to the increase in MIC of ceftriaxone observed in 2022–2023 (**Figure 1A**).

**Table 1:** Distribution of genetic determinants of *N. gonorrhoeae* resistance to ceftriaxone and azithromycin in the Russian population (2015-2023).

Analyzed locus	Detected determinants	Percentage of isolates (%)							
		2015	2016	2017	2018	2019	2020	2021	2022-2023
*penA*	Ala311 (WT)	100	100	100	100	100	100	100	100
	Val311	0	0	0	0	0	0	0	0
	Ile312 (WT)	98	100	98	97	98	97	91	89
	Met312	2	0	2	3	2	3	9	11
	Val316 (WT)	98	100	98	97	98	97	91	89
	Thr316	2	0	2	3	2	3	9	11
	No ins345-346 (WT)	24	34	16	27	35	20	24	21
	InsAsp345-346	76	66	84	73	65	80	76	79
	Ala501 (WT)	99	96	84	97	97	91	89	98
	Val501	1	0	1	1	3	9	11	2
	Thr501	0	4	15	3	0	0	0	0
	Asn512 (WT)	98	100	98	96	98	97	91	89
	Tyr512	2	0	2	4	2	3	9	11
	Gly542 (WT)	67	83	57	76	82	88	67	74
	Ser542	33	17	43	24	18	12	33	26
	Gly545 (WT)	98	100	98	96	98	97	91	90
	Ser545	2	0	2	4	2	3	9	10
	Pro551 (WT)	98	95	95	94	97	97	93	98
	Ser551	1	0	1	1	2	3	4	2
	Leu551	1	5	4	5	1	0	2	0
	Mosaic sequence	2	0	2	3	2	3	9	11
*ponA*	Leu421 (WT)	60	68	46	57	70	78	52	63
	Pro421	40	32	54	43	30	22	48	37
*porB*	Gly120 (WT)	74	78	67	87	87	72	54	89
	Lys120	7	12	22	7	6	26	39	8
	Asn120	1	2	1	0	0	0	0	0
	Asp120	18	6	10	6	7	3	7	0
	Arg120	0	0	0	0	0	0	0	3
	Thr120	0	1	0	0	0	0	0	0
	Ala121 (WT)	88	84	64	92	90	66	41	81
	Asp121	2	9	19	1	1	3	9	5
	Asn121	2	4	2	3	0	22	35	6
	Ser121	0	0	2	0	0	7	11	8
	Gly121	7	4	13	3	9	1	4	0
*mtrR* (prom.)	-35 (WT)	82	80	66	83	89	76	65	61
	-35 delA	18	20	34	17	11	24	35	39
	-35 (MG-like)	0	0	0	1	1	15	22	19
	-35 (WHOP-like)	0	0	0	0	0	0	2	0
	-10 (WT)	100	100	100	99	100	100	100	100
	-10 insT/insTT	0	0	0	1	0	0	0	0
*mtrD*	Ser821/Lys823 (WT)	100	100	100	99	98	82	63	68
	Ala821/Glu823 (mosaic)	0	0	0	1	2	18	37	32
23S rRNA	C2611 (4/4 alleles) (WT)	100	100	100	100	100	98	100	98
	C2611T (4/4 alleles)	0	0	0	0	0	2	0	2
**Total number of isolates for each year**		123	253	127	150	119	116	46	62

Total number of isolates for each year (actual values).

The increase in the number of azithromycin-resistant isolates in Russia in 2020 is linked to the emergence of mosaic alleles in the *mtrR* (meningitidis-like promoter) and *mtrD* genes. From 2021 to 2023, the proportion of isolates with the meningitidis-like promoter in the *mtrR* gene, as well as the Ala821/Glu823 substitutions in the *mtrD* gene, remained stable, suggesting that these genetic determinants have become fixed in the population. Consequently, resistance to azithromycin is likely to be sustained. It is also noteworthy that only three isolates with mutations in the 23S rRNA gene (2611 position in all 4 copies) were detected during the entire study period. These isolates, which are associated with resistance to azithromycin (MIC ≥ 4 mg/L), were found sporadically in the population.

During the analyzed period, the proportion of isolates with Pro421 mutations in the *ponA* gene and with Lys120, Asn120, Thr120, and Asp121 substitutions in the *porB* gene remained quite stable, with some exceptions. However, the proportion of isolates with Asp120 and Gly121 mutations in the *porB* gene has markedly decreased, while the percentage of isolates with Arg120, Asn121, and Ser121 mutations has generally increased. This shift is likely due to changes in both *porB* alleles and the molecular genotypes circulating in Russia.

### Genotypes of *N. gonorrhoeae* isolates

Between 2015 and 2023, 325 different NG-MAST sequencing types and 80 MLST types were identified in the analyzed sample. A complete list of the NG-MAST and MLST types for all samples analyzed is provided in **Supplementary Table S1**. For NG-MAST, nucleotide distances were calculated, and genetic groups were established. Of the 325 NG-MAST sequencing types, 222 formed 48 distinct genetic groups, while the remaining 103 types were classified as ungrouped STs. The major Russian *N. gonorrhoeae* genogroups are summarized in **Table 2**.

**Table 2:** Distribution of 996 Russian *N. gonorrhoeae* isolates by the most frequently observed genogroups and linked MLSTs.

Genogroup	Number of isolates (%)	NG-MAST (Number of isolates, %)	Linked MLST (Number of isolates, %)
G807	222 (22.0%)	807 (36.0%); 228 (17.1%); 1544 (7.2%); 9570 (5.9%); 9576 (3.2%); 5941 (3.2%); 8901 (2.7%); 13054 (2.7%); 18894 (2.3%); 13316 (2.3%); 24556 (2.3%); 4570 (1.4%); 18246 (0.9%); 12555 (0.9%); 3369 (0.9%); 12529 (0.9%) 16177 (0.9%); 14626 (0.9%); 12557 (0.9%); 3321 (0.9%); 24571 (0.9%); 14005 (0.5%); 13059 (0.5%); 13456 (0.5%); 14624 (0.5%); 14606 (0.5%); 1531 (0.5%); 14008 (0.5%); 15619 (0.5%); 6967 (0.5%); 20267 (0.5%); 14935 (0.5%); 24566 (0.5%); 14024 (0.5%)	1594 (91.9%); 13757 (3.6%); 1892 (1.4%); 1901 (1.4%); 7371 (0.5%); 1579 (0.5%); 11704 (0.5%); 17601 (0.5%)
G1993	108 (10.8%)	1993 (38.9%); 5714 (27.8%); 14006 (6.5%); 14627 (5.6%); 16411 (4.6%); 17027 (3.7%); 16174 (2.8%); 14625 (1.9%); 14628 (1.9%); 20273 (1.9%); 18247 (0.9%); 13061 (0.9%); 14612 (0.9%); 14610 (0.9%); 9660 (0.9%)	11177 (82.4%); 1901 (6.5%); 1579 (2.8%); 1594 (2.8%); 1892 (1.9%); 14009 (1.9%); 14947 (1.9%)
G9574	41 (4.1%)	9574 (19.5%); 5042 (17.1%); 12449 (17.1%); 14940 (7.3%); 13459 (7.3%); 12444 (7.3%); 15748 (4.9%); 17020 (4.9%); 14939 (2.4%); 9569 (2.4%); 20292 (2.4%); 2873 (2.4%); 14932 (2.4%); 12600 (2.4%)	1892 (73.2%); 1901 (19.5%); 1594 (4.9%); 1588 (2.4%)
G13058	36 (3.6%)	13058 (36.1%); 6226 (30.6%); 13055 (27.8%); 16183 (2.8%); 17026 (2.8%)	1901 (100%)
G12302	32 (3.2%)	12302 (28.1%); 17380 (18.8%); 14502 (9.4%); 20602 (9.4%); 16953 (6.2%); 18787 (6.2%); 20279 (3.1%); 20291 (3.1%); 3935 (3.1%); 20275 (3.1%); 8241 (3.1%); 20294 (3.1%); 20274 (3.1%)	9363 (96.9%); 15183 (3.1%)
G1152	24(2.4%)	1152 (16.7%); 387 (12.5%); 17017 (8.3%); 14027 (8.3%); 5734 (8.3%); 14600 (8.3%); 14825 (8.3%); 12560 (4.2%); 5711 (4.2%); 17532 (4.2%); 13455 (4.2%); 2678 (4.2%); 5185 (4.2%); 24564 (4.2%)	1892 (66.7%); 7365 (12.5%); 8135 (8.3%); 13757 (4.2%); 7371 (4.2%); 11177 (4.2%)
G2212	20 (2.0%)	2212 (20.0%); 10025 (20.0%); 4706 (20.0%); 15212 (10.0%); 20283 (5.0%); 1407 (5.0%); 3149 (5.0%); 20278 (5.0%); 5622 (5.0%); 22865 (5.0%)	1901 (50.0%); 1902 (20.0%); 8137 (10.0%); 16272 (5.0%); 16267 (5.0%); 15273 (5.0%); 17540 (5.0%)
G4707	12 (1.2%)	4707 (33.3%); 972 (25.0%); 14013 (8.3%); 14614 (8.3%); 3329 (8.3%); 24569 (8.3%); 14619 (8.3%)	1901 (100%)
Other	300 (30%)	–	–
Ungrouped	201 (20%)	–	–

MLST genotypes were determined for all isolates within the major NG-MAST genogroups (**Table 2**). Notably, in most cases, isolates within a single NG-MAST genogroup shared a dominant MLST genotype. As a result, a strong association between NG-MAST genogroups and MLST types was observed, with approximately 80% of isolates in the major genogroups in the Russian population exhibiting this linkage. The exception was genogroup G2212, where 50% of isolates had MLST 1901 and 20% had MLST 1902, accounting for a total of 70% of the genogroup.

The most common genogroup, G807, includes 222 isolates (22% of all samples), representing 34 STs. This genogroup encompasses the three most frequently occurring NG-MASTs in the Russian Federation: ST807, ST228, and ST1544. Among all isolates in genogroup G807, MLST 1594 was detected in 91.9% of isolates. The second most common genogroup in Russia, G1993, consists of 108 isolates (10.8%) belonging to 15 sequence types, with 82.4% of isolates linked to MLST 11177.

The genogroups G2212, linked to MLST 1901/1902 (accounting for 70% of isolates), and G12302, linked to MLST 9363 (96.8% of isolates), pose the greatest threat to the Russian population. Isolates of genogroup G2212 represented 2% of the sample and were most commonly associated with a mosaic structure in the *penA* gene, leading to decreased susceptibility to ceftriaxone, as previously reported (Kandinov et al., 2020). Genogroup G12302 comprised 3.1% of the sample, and its emergence in Russia in 2020 was linked to the rise of azithromycin-resistant isolates carrying mosaic alleles in the *mtrR* and *mtrD* genes (Kandinov et al., 2023b).

**Figure 2:**
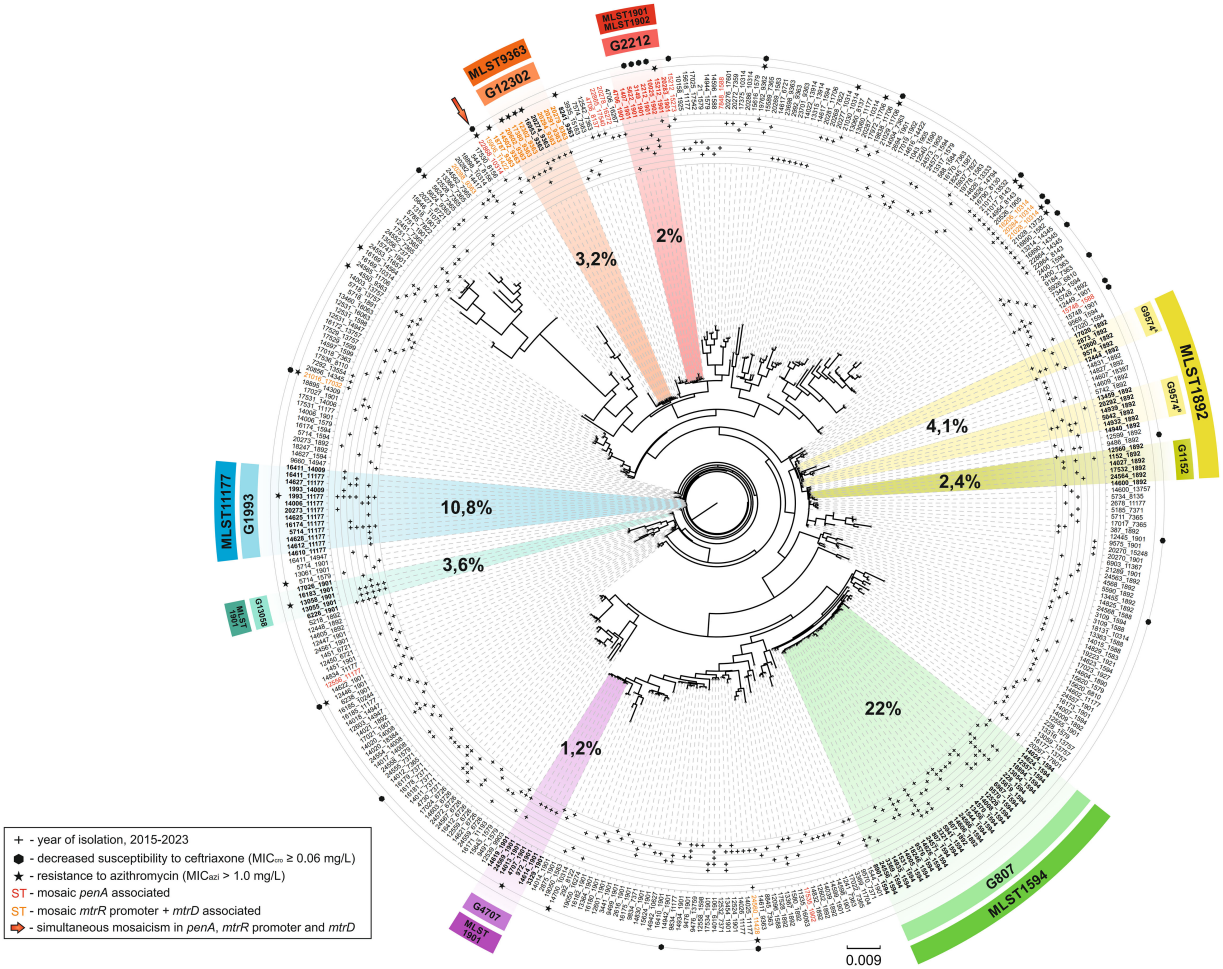
Maximum likelihood phylogeny of NG-MAST and MLST types for *N. gonorrhoeae* isolates collected in Russia (2015-2023). The tree consists of 376 terminal nodes, each representing a unique combination of NG-MAST and MLST types (labeled as node names). The years in which a clade is found are marked by ‘plus’ signs and arranged from 2015 to 2023 in increasing radius order. Clades associated with reduced susceptibility to ceftriaxone and/or resistance to azithromycin are marked by ‘hexagon’ and ‘star’ signs, respectively. Colors on the tree correspond to the main NG-MAST genogroups in the Russian population, along with the MLST types linked to each genogroup. The percentage share of each genogroup in the total sample is indicated in bold black font. Sequence types associated with mosaic *penA*, as well as *mtrR* promoter + *mtrD*, are highlighted in red and orange font, respectively. The sequence type with both mosaic *penA* and *mtrR* promoter + *mtrD* is indicated by an arrow.

A maximum likelihood phylogenetic tree was constructed for the NG-MAST and MLST types of *N. gonorrhoeae* isolates collected in Russia from 2015 to 2023 (**Figure 2**). The tree reflects a total of 376 unique NG-MAST + MLST combinations present in the Russian population of *N. gonorrhoeae*.

The phylogeny of the Russian *N. gonorrhoeae* population demonstrates a clear linkage between NG-MAST genogroups and MLST types. Notably, some major genogroups are located relatively close to one another on the tree. For example, genogroup G9574 is split into two clusters, G9574A and G9574B, and is positioned near genogroup G1152. The majority of isolates within this genogroup are associated with MLST 1892, suggesting a common ancestral origin for these genogroups. Genogroups G1993 and G13058 are also in close proximity, although they are linked to different MLST types, 11177 and 1901, respectively.

The pandemically significant genogroups G2212 and G12302 are phylogenetically close but linked to distinct MLST types, 1901/1902 and 9363, respectively. Interestingly, both of these genogroups are positioned at a considerable distance from the dominant Russian genogroup, G807.

### Association of mosaic variants of *penA*, *mtrR*, *mtrD* genes with molecular genotypes

**Figure 3:**
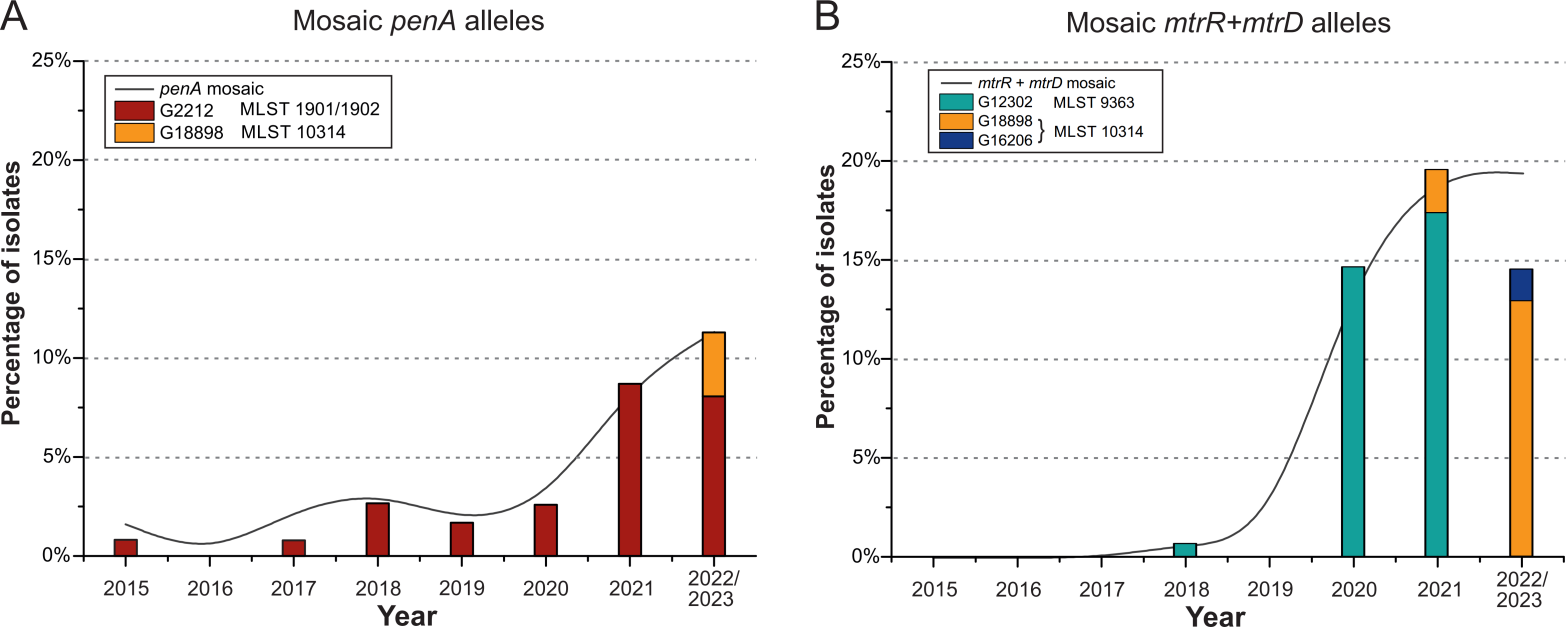
Distribution of the proportion of *N. gonorrhoeae* isolates by NG-MAST genogroups and MLST in the Russian population (2015-2023). **(A)** Isolates with mosaic structure of the *penA* gene. **(B)** Isolates with mosaic structure of the *mtrR* (promoter) and *mtrD* genes. The black line represents the percentage of isolates with the specified mosaic gene structures. The colored bars indicate the number of isolates belonging to different NG-MAST genogroups and MLST types.

From the total sample of isolates collected between 2015 and 2023, we selected those in which mosaic structures in the *penA* gene (24 isolates) and simultaneous mosaicism in the *mtrR* (promoter) and *mtrD* genes (40 isolates) were detected. The distribution of these isolates, based on the year of collection, is shown in **Figure 3**.

As shown in **Figure 3A**, between 2015 and 2021, *penA* mosaic alleles were predominantly found in isolates from genogroup G2212. However, in the 2022-2023 sample, *penA* mosaicism was also detected in isolates from genogroup G18898. Notably, isolates from genogroup G18898 are associated with MLST 10314, in contrast to G2212, which is linked to MLST 1901 and 1902.

In 2020 and 2021, mosaicism in the *mtrR* (promoter) and *mtrD* genes was primarily associated with genogroup G12302 and MLST 9363 (**Figure 3B**). Notably, no isolates from this genogroup were detected in the 2022-2023 sample. Instead, in the 2022-2023 isolates, mosaicism in the *mtrR* (promoter) and *mtrD* genes was linked to the novel genogroups G18898 and G16206, both associated with MLST 10314.

For the first time, isolates with simultaneous mosaicism in the *penA*, *mtrR* (promoter), and *mtrD* genes were identified in the Russian population. A total of two isolates exhibiting simultaneous mosaicism in all three genes were found in the 2022–2023 sample. Both isolates had an MIC_cro_ of 0.03 mg/L and MIC_azi_ of 1 mg/L and 2 mg/L (the second isolate is resistant according to EUCAST criteria). Thus, a new cluster of isolates from genogroup G18898 and MLST 10314 was identified in Russia, which may lead to the development of simultaneous resistance to both ceftriaxone and azithromycin in the near future.

### Forecast of the distribution of isolates with mosaic genes *penA*, *mtrR*, *mtrD* in the population

**Figure 4:**
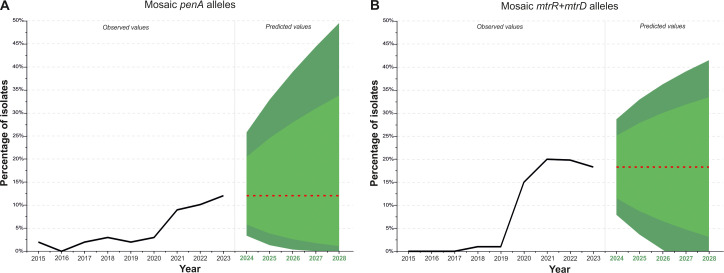
Dynamics of isolates with mosaic *penA* **(A)** and mosaic *mtrR* (promoter) + *mtrD* **(B)** in the population, with predictions for the next 5 years (2024-2028). The black line represents the percentage of isolates obtained experimentally between 2015 and 2023. The red line indicates projections for the next 5 years. The light green area represents the 80% confidence interval, while the dark green area represents the 95% confidence interval.

To better understand the processes within the current Russian population of *N. gonorrhoeae* and to assess possible future trends, we predicted the distribution of isolates with mosaic *penA* genes, as well as those with simultaneous mosaicism in the *mtrR* promoter and the coding region of the *mtrD* gene. Parameter selection was performed for ARIMA models describing the dynamics of genotypes with mosaic *penA* and with mosaic *mtrR* (promoter) and *mtrD*. In both cases, the best models were of the ARIMA (0,1,0) class, predicting the spread of the corresponding genotypes as a random walk around the trend. The analyzed time series and predictions for the occurrence of *penA* (A) and *mtrR* (promoter) and *mtrD* (B) mosaic genotypes in the Russian population are shown in **Figure 4**.

Analysis of the frequencies of genotypes with mosaic *penA* (**Figure 4A**) shows that, in 2021, there was an increase in the proportion of isolates to 9%, compared to previous years when the frequency of mosaic *penA* was around 2–3%. The frequency of isolates with the mosaic *penA* gene remained above 9% after 2020 and is projected to stabilize at approximately 12% between 2024 and 2028, accounting for possible fluctuations. Even under the most favorable conditions, within the 80% confidence interval, gene drift is unlikely to reduce the proportion of mosaic *penA* isolates to 1% or less in the next few years. In the case of unfavorable developments, with an increase in the proportion of mosaic *penA*, the 80% confidence interval suggests that the proportion of isolates with mosaic *penA* will not exceed one-third of all isolates.

Investigation of the frequency of isolates with simultaneous mosaicism in the *mtrR* (promoter) and *mtrD* genes (**Figure 4B**) revealed a sharp increase in the proportion of isolates in 2020, with the frequency remaining consistently high at around 18% between 2020 and 2023. The forecast for 2024–2028 predicts values near 18%. Within the 80% confidence interval, it can be concluded that the proportion of isolates with mosaic *mtrR* and *mtrD* genes is unlikely to decrease to 3% or less, considering possible fluctuations. In the case of unfavorable developments, the proportion of genotypes with mosaic *mtrR* and *mtrD* genes may increase to one-third of all isolates.

## Discussion

In this study, we investigated the structure of the Russian *N. gonorrhoeae* population, including susceptibility to ceftriaxone and azithromycin, and the molecular characteristics of 996 isolates collected in Russia between 2015 and 2023. No ceftriaxone-resistant isolates were detected, confirming the continued feasibility of using this antimicrobial in treatment regimens. Among the study samples, the proportion of isolates with reduced susceptibility to ceftriaxone did not exceed 1–2% per year between 2015 and 2021. It should be noted separately that reduced susceptibility to ceftriaxone in the Russian population of *N. gonorrhoeae* may not always be associated with a single specific determinant and often exhibits a polygenic nature. The combination of mutations in the *penA*, *ponA*, *porB*, and *mtrR* genes, as well as other unstudied spontaneous mutations in the genome and mechanisms, may be the reason for the demonstrated slow decline in susceptibility to ceftriaxone over the past few years. However, in 2022–2023, 22.6% of isolates showed reduced susceptibility, which may signal a worsening of the epidemiological situation in the near future.

Despite the fact that azithromycin is not recommended for the treatment of gonococcal infections in Russia, approximately 12% of isolates resistant to this antimicrobial agent, according to EUCAST criteria, have been registered annually since 2020. This is higher than the WHO-recommended threshold of 5%. The persistence of a high proportion of azithromycin-resistant isolates in Russia rules out the use of the “ceftriaxone + azithromycin” combination therapy, which is used in some other countries (WHO, 2024b; Golparian et al., 2024). Overall, Russia still has a relatively favorable epidemiological situation compared to Europe, Asia, and the USA, where both multidrug-resistant (MDR) and extensively drug-resistant (XDR) strains are regularly detected (Maubaret et al., 2023; Tang et al., 2023; Ouk et al., 2024).

Oligonucleotide microarrays remain a reliable tool for the identification of genetic determinants of resistance to current antimicrobials, as well as mass genotyping of clinical isolates of *N. gonorrhoeae*. In this work, using previously developed microarrays (Shaskolskiy et al., 2021a; Kandinov et al., 2023a, 2024), we obtained data on the distribution of genetic determinants of resistance of *N. gonorrhoeae* to ceftriaxone and azithromycin on the territory of the Russian Federation in 2015-2023.

The dynamics of isolates with mosaic alleles of the *penA*, *mtrR*, and *mtrD* genes are of particular interest in the analyzed sample. Previously, we identified mosaic alleles of the *penA* gene (type 34.001) in Russian isolates with reduced susceptibility to ceftriaxone (Kandinov et al., 2020). This study shows an increase in the proportion of isolates with mosaic *penA* to 9% in 2021, and to 11% in 2022–2023, compared to previous years, when the proportion of such isolates did not exceed 2–3%. These findings indicate not only the consolidation of isolates with mosaic *penA* alleles in Russia, but also a gradual increase in the proportion of such isolates in recent years.

Isolates with the *mtrR* mosaic promoter (alleles 485 and 530 on NG-STAR) and the *mtrD* mosaic promoter (allele 3353 on pubMLST) were first detected in Russia in 2020 (Kandinov et al., 2023b). In the present study, analysis of isolates from 2022–2023 revealed a consistently high frequency of these isolates, indicating their establishment in the population.

Determinants not associated with mosaic alleles in *penA*, *ponA*, *porB*, *mtrR*, *mtrD*, and 23S rRNA genes remained relatively stable throughout the study period, with some exceptions. Isolates with C2611T mutations in the 23S rRNA gene and isolates with a WHO-P mosaic promoter in the *mtrR* gene were sporadically detected. The appearance or disappearance of other determinants in the studied genes appears to be largely associated with changes in molecular genotypes in Russia.

Eight major genetic groups of NG-MAST were detected in Russia. Isolates from genogroups G807 and G1993 were the most common, accounting for 22% and 10.8% of the samples, respectively. Genogroups G9574, G13058, G12302, G1152, G2212, and G4707 were less frequent, with the remaining genogroups comprising 30% of the total sample, and 20% of isolates remained ungrouped. The phylogenetic analysis of all identified NG-MAST and MLST types in Russia revealed a strong association between NG-MAST types within a genogroup and a specific MLST type. For example, all isolates in NG-MAST genogroup G807 were associated with MLST 1594 in 91.9% of cases, isolates from G1993 were linked to MLST 11177 in 82.4%, and so on. Overall, 80% of isolates in the sample showed a clear linkage between NG-MAST genogroups and MLST types.

In 2015, isolates from the pandemically significant genogroup G2212 (known globally as G1407) were detected in Russia for the first time (Kubanov et al., 2016). This genogroup has been detected annually throughout the study period and continues to increase in frequency. It is important to note that isolates from this genogroup exhibit reduced susceptibility to ceftriaxone due to the presence of the mosaic *penA* 34.001 type. The second genogroup of concern for Russia is G12302, first identified in the Russian Federation in 2020, which is associated with azithromycin resistance due to the presence of mosaic *mtrR* (promoter) and *mtrD* genes (Kandinov et al., 2023b). We previously expressed concern that the presence of these two genogroups in Russia could lead to a worsening of the epidemiological situation (Kandinov et al., 2023a). In the present study, we characterized two additional genogroups, G18898 and G16206 (both linked to MLST 10314), in the 2022–2023 sample. These genogroups were found to contain isolates with mosaic *penA* alleles, either alone or in combination with mosaic *mtrR* (promoter) and *mtrD*.

For the first time, two isolates with simultaneous mosaicism of *penA*, *mtrR* (promoter), and *mtrD* were detected. Both isolates belonged to the NG-MAST genogroup G18898 and MLST 10314. MLST 10314 has been previously identified globally, including in China, the UK, and other countries, and isolates with this genotype often carry the *penA* mosaic type 60.001, which is associated with reduced susceptibility or resistance to third-generation cephalosporins (Maubaret et al., 2023; Tang et al., 2023). Notably, G18898 and MLST 10314 have been present in Russia since 2017 (Shaskolskiy et al., 2020), but earlier isolates from this genogroup did not carry mosaic alleles.

The observed sharp changes in the proportions of isolates with mosaic *penA* and with mosaic *mtrR* promoter and *mtrD*, which occurred in 2021 and 2020 respectively, led to the fixation of the above-mentioned genetic determinants of resistance in the population. The prediction made by the time series analysis suggests that isolates with mosaic alleles will not be eliminated from the population. In case of unfavorable development of events, it is likely that in 5 years every third isolate will have mosaicism of either *penA* or *mtrR* and *mtrD* simultaneously. Successful fixation of G18898 and MLST 10314 in Russia will inevitably lead to deterioration of the epidemiological situation in the country.

In summary, the autochthonous genetic types prevalent in the Russian population of *N. gonorrhoeae*, which are susceptible to both cephalosporins and macrolides, are gradually being replaced by globally dominant genetic lineages. Over the past three years, genetic determinants of reduced susceptibility to both macrolides and cephalosporins have become firmly established in the population. Ongoing surveillance, including molecular epidemiology methods and the collection of data on genetic markers of antimicrobial resistance, will play a crucial role in curbing the growth and spread of *N. gonorrhoeae* resistance, preventing further dissemination, and ensuring the continued availability of effective treatment options.

## Data Availability

The original contributions presented in the study are included in the article/**Supplementary Material**. Further inquiries can be directed to the corresponding author.
